# Contingency Planning and Early Crisis Management: Italy and the COVID-19 Pandemic

**DOI:** 10.1007/s11115-021-00545-1

**Published:** 2021-08-31

**Authors:** Paola Mattei, Lorenzo Vigevano

**Affiliations:** Department of Social and Political Sciences, University of Milan, Milan, Italy

**Keywords:** Contingency planning, Early crisis management, COVID-19, Italy’s pandemic plans, Policy integration

## Abstract

This study examines the level of preparedness exhibited through strategy, planning and organization to deal with the COVID-19 pandemic in Italy. A comparative analysis of four regions revealed that the implementation of pandemic plans was affected by multiple factors. For instance, some planning was outdated and insufficient to cope with the new threat posed by the pandemic; due to a decentralized health care system, there was confusion about whether regional or national decision-making was the coordinating actor; shortages in supplies and equipment such as masks, in some regions, were due to lack of implementation of existing pandemic plans. The study emphasizes the importance of a coordinated response to crises.

## Introduction

Over 163 million people, worldwide, have been affected by the Coronavirus disease (COVID-19) since January 2020 (WHO Coronavirus Dashboard). This new, extraordinarily contagious strain of Severe acute respiratory syndrome (SARS) virus persuaded several national governments to implement radical policy measures to alleviate its catastrophic impact on the population. Its effects were profoundly disruptive and unpredictable: no health care system, globally, proved ready to cope with such an overwhelming volume of infections, rapidity of contagion, and alarming death toll. Most health care systems faced severe capacity overloads, and significant losses of lives followed where the outbreaks occurred. By the end of April 2021, the total deaths, globally, surpassed 3.28 million (WHO Coronavirus Dashboard).

Originating from the Wuhan province in China, when the virus reached Europe, even the high-performing Italian Health Care System proved unable to manage it (Mattei & Del Pino, 2021). The *Servizio Sanitario Nazionale* was the first European National Health Service (NHS) provider to be intensely affected by the virus; hence, it was impossible to build upon previous or proximate policy experiences. Furthermore, the provision of health care services is fragmented due to a strongly decentralized governance structure. Since the reform of health care services in 1992, Italian regions enjoy substantial autonomy in planning, organizing, financing, and delivering health care services (Mattei, 2006) according to their respective health care crisis management strategies. Moreover, locally adopted mechanisms for pandemic preparedness coincided, only partially, across the national territory, leading to highly heterogenous approaches to emergency response and criticalities regarding coordination with the central government.

The key outcome variable in this paper is the level of pre-crisis pandemic preparedness and anticipatory planning instruments put in place at national and regional scales to manage pandemics. How did regional governments implement their pandemic plans, as key policy instruments designed to enhance policy coordination and integration? The main hypothesis, in this paper, is that in a highly decentralized governance system, joint actions and collaboration between interdependent actors and levels of government is crucial for crisis management, and this emerged as the most significant governance criticality from early policy responses to COVID-19 in Italy.

This paper focuses on early interventions and contingency planning for the transboundary COVID-19 crisis in Italy, for the “first wave” pandemic period spanning January to June 2020. The study’s aim is to understand the country’s level of preparedness to deal with the pandemic. Planning in advance, and preparation through useful policy instruments, protocols, and definitions of responsibilities between different levels of government and stakeholders allows efficient responses during crisis management and swift applications of regulations and procedures.

Typical issues arising in transboundary crises are so called “wicked problems,” in which crisis management efforts of multiple interdependent authorities, with varying degrees of autonomy, are confused by the unique, indefinite, and multidimensional nature of the emergencies (Rittel & Webber, 1973). “Wicked problems” can disrupt governance capacity, especially when policy actors are unable to harmonize emergency responses due to unclear hierarchies and remits. For this study’s analysis, the organizational character of the governance system for crisis management, and its relative ability to coordinate a plurality of actors, constitutes a cardinal explanatory factor of the system’s capacity to deal with the pandemic crisis in an effective way.

During crises, policy making occurs in a context of extreme uncertainty, under time urgency, and enormous public pressure as citizens facing a major threat expect political leaders to minimize the impact of the crisis, to establish a sense of normality, and foster collective learning (Boin et al., 2016). A degree of political risk is implied in all such processes, as decisionmakers are assessed according to the public’s perception of performance regarding crisis management. Lodge and Weigrich identify an organizational domain that is important to this study’s analysis: the capacity to coordinate policy between various levels of government and across overlapping policy sectors (2014). The concurrent intervention of government-funded agencies, specialized organizations, and local authorities are presumed preferable to ministerial intervention due to greater proximity to the operational level (Christensen et al., 2016), but increases the risk of coordination failures (Christensen & Laegreid, 2007; Lagreid & Rykkja, 2015).

This qualitative study incorporates empirical data that outline anticipatory measures to deal with pandemics at national and regional government levels. Documents produced before the COVID-19 pandemic emerged, that is, before January 2020, were collected and analyzed. The study focused on policy documents and pandemic plans that were formally and legally binding for relevant public authorities, and on administrative regulations and legislation. The 2006 National Pandemic Plan (NPP) and selected regional plans are statutory documents publicly available and easily downloadable from the government website. Categories and themes were developed by analyzing the planning documents inductively. These were evaluated across different regional perspectives. A comparison was then made between the regional approaches and the 2006 NPP. The dimensions of comparison, which were derived inductively, are as follows:

- Stockpiling of Personal Protective Equipment (PPE).
- Training of a health care workforce, including doctors and health workers.
- Roles given to communication strategies at a regional level.

The study does not intend to make generalizations but highlights similarities and differences across regional approaches and how selected regions have implemented the national plan (Ragin, 2014). In addition to administrative regulations and legislation, government ministers’ statements, government press releases, guidance for doctors, and emergency executive orders were collected and studied to analyze the contexts within which these plans were designed and adopted.

This article consists of three sections. “Theoretical Framework” section introduces and discusses the concepts of transboundary crisis, underlining this study’s proposition that performance in crisis management fundamentally lies in governance capacity and coordination, especially in decentralized systems. “Context: Italy” section examines the context of institutional and organizational characteristics of decentralized health care systems to understand the Italian case. Meanwhile, “Pandemic Plans” section presents the national and regional implementation strategies of plans to cope with the pandemic. The conclusive discussion in “Concluding Discussion” section surmises the main empirical findings and frames the contributions of the study to advance the scholarly debate on the management of transboundary and health care crises for decentralized governance systems.

## Theoretical Framework

The absence of any planning is usually a recipe for chaos and confusion, and frequently results in crisis mismanagement (Eriksson & McConnell, 2017). In decentralized governance systems, crisis planning is important to facilitate collaboration between the central, regional, and local levels of government, for joint action to achieve the goal of virus containment and control. Moreover, coordination is emphasized as a determinant of government’s ability to achieve a shared definition of the problem, structure a feasible, effective, and acceptable course of action, effectively present it to the public, allocate the resources required, and assign crisis-ownership through the harmonization of multiple levels of actors and organizations in a collective, yet directed, effort (Weick, 1995).

Existing literature on transboundary crises and its management offers useful analytical tools to explain how national governments respond. Transboundary crises are marked by unprecedented levels of uncertainty and critical threat to citizens, and COVID-19 fits all five characteristics developed by Boin et al. (2016). First, it affects multiple policy domains; not merely one sector of government activities. Second, the crisis developed rapidly, and national governments had to respond and design measures under unprecedented time pressure. Third, it was very difficult to comprehend what was happening in the initial stages of the COVID-19 outbreak, as it originated in a faraway country and the causes and origins of the outbreak were unknown and contested for a long time. Fourth, the COVID-19 crisis posed huge challenges to the existing bureaucratic and political system, and multiple actors at different levels of government, regarding accountability and responsibility. Fifth, no ready-made, easily applicable solution to the COVID-19 emergency was immediately available to policymakers. In Italy, the crisis proved extremely demanding for its unpredictably pervasive impact on multiple organizational domains on one hand, and due to its uniqueness and complexity on the other.

Crisis management can be approached by focusing on governance-centered strategies and examining the framing of policy processes, and implementation issues. It can be viewed also from the theoretical lenses of government-centered approaches focusing on organizational coordination and structural dimensions of administrative units (Christensen et al., 2016). This section presents both approaches, given the interconnectedness of policy making with institutional determinants. However, this paper leans towards the governance approach, with a focus on policy instruments implemented by regional governments for contingency planning. Policy capacity has been defined as the government’s ability to conduct an array of policy-related functions, pertinent to three distinct domains (Wu et al., 2015): the capacity to understand the policy environment, to identify critical actors and assets, and to provide strategic guidance. With the novel COVID-19, obtaining a quick grasp of the situation turned into an insurmountable endeavor due to the rapid escalation of the numbers of people infected, the medical conditions in lethal cases, and the initial epidemiological patterns of contagion. Although national and regional pandemic plans were in place, as discussed later, entire governance systems were overwhelmed by the pandemic.

As the COVID-19 crisis arose from the transboundary space, existing structural governance arrangements to manage such an occurrence were fundamental. The decentralized structure of health care governance in Italy is thus particularly relevant (Saltman et al., 2007). Bringing together disparate organizations at central and subnational levels to engage in coherent and joint action during a transboundary crisis is particularly challenging in analogous systems. Coordination is defined as “adjustment of actions and decisions among interdependent actors to achieve a specific goal” (Koop & Lodge, 2014) and is considered a mechanism for governments to manage “wicked” policy problems. Ansell suggested that in large-scale transboundary crises, coordination among actors may present a significant challenge. The larger the scale of the crisis, the higher the number of actors involved; hence, it becomes increasingly difficult to decide, unanimously, which public authority is to lead the coordination effort through accepted enforcement mechanisms (Ansell et al., 2010). The inability to match the organizational structure of intervention with the multiple dimensions of the crisis creates policy underlaps, where two or more authorities sharing different degrees of competence over the same domain expect the other to intervene. As neither actor can clearly frame its own responsibility in the context of the emergency, intervention may be foregone, delayed, or enfeebled. In the Italian system, responsibility for containment measures are devolved to regional governments, although occasions for centralized intervention are envisioned in preventive pandemic plans.

**Fig. 1:**
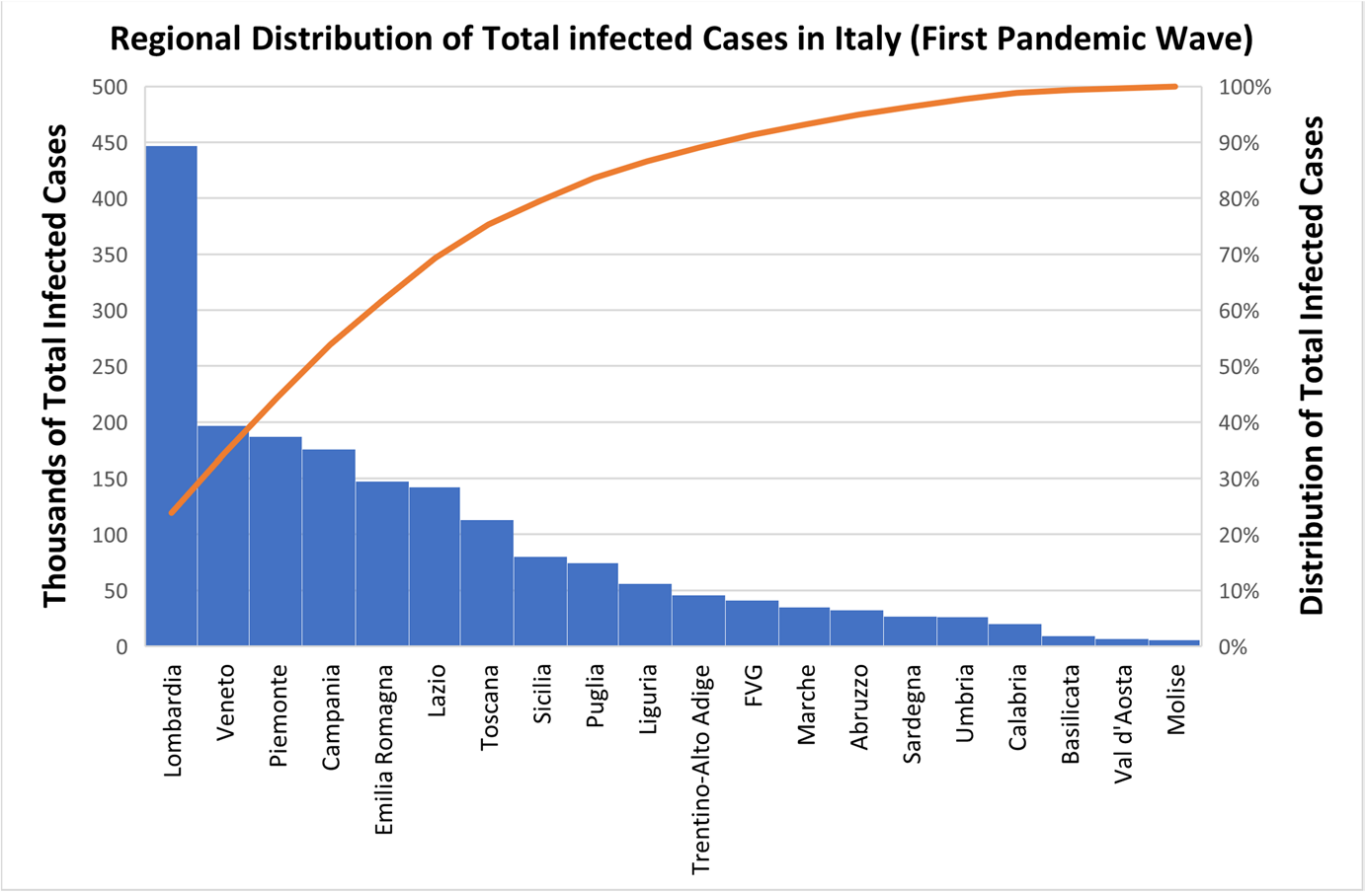
Bars represent regional total cases in thousands; the line represents the compounded distribution of total cases from most to least affected regions. *Source* Ministero della Salute & Istituto Superiore di Sanità (2020)

The first wave of the COVID-19 crisis was characterized by extreme territorial localization. Figure 1 highlights the marked concentration of the disease in the region of Lombardy up to the first pandemic wave. The effect in Lombardy was remarkably severe compared to other areas, including neighboring regions such as Emilia Romagna, or Piemonte.

**Fig. 2:**
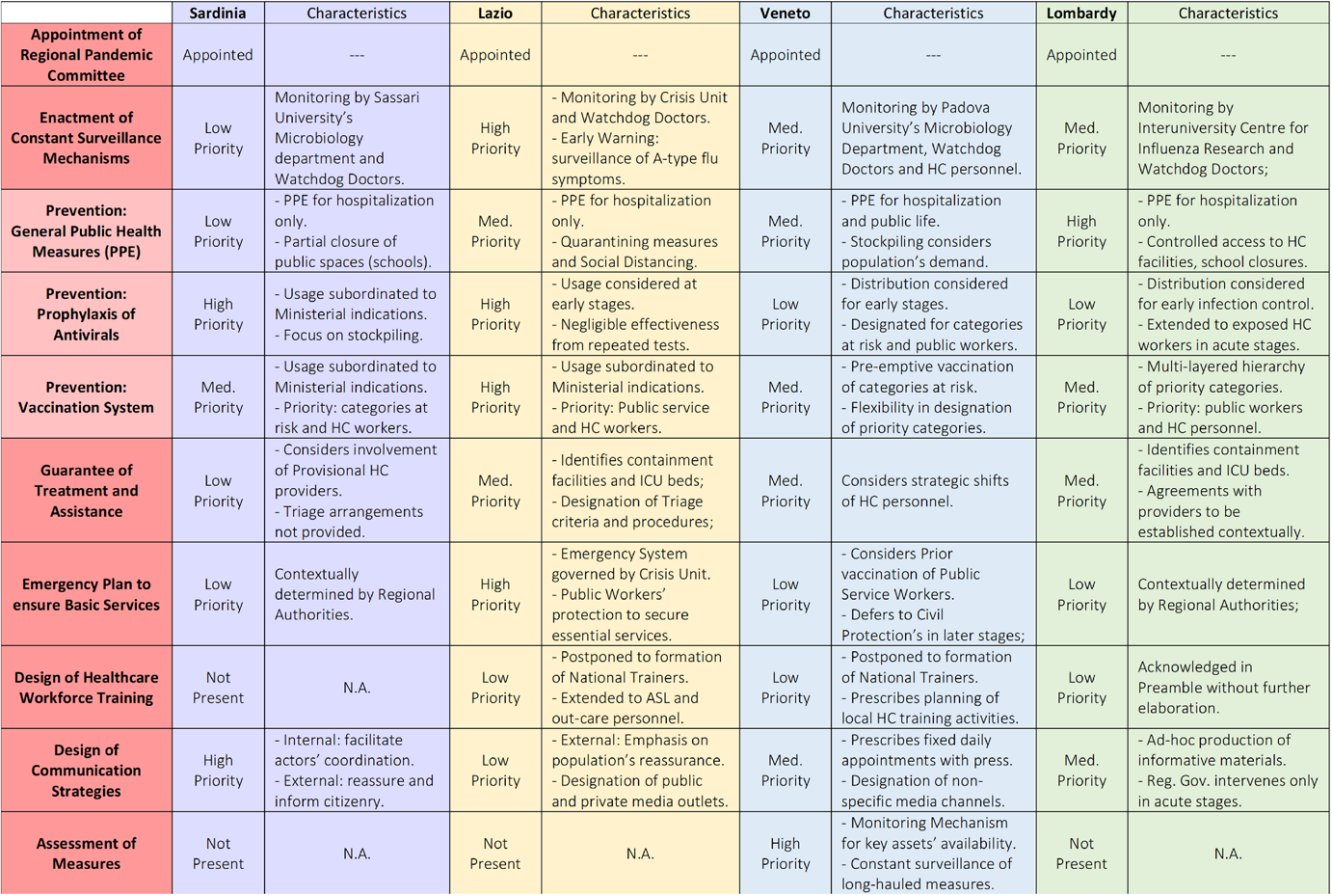
Key characteristics of Regional Pandemic Plans for selected regions. *Source* Consiglio regionale della Lombardia (2006), Giunta regionale del Lazio (2008), Ministero della Salute (2006) and Regione Autonoma della Sardegna (2009)

Crisis-management instruments and responsibilities do not flow exclusively in a vertical fashion from higher levels of government to regional and local authorities. Horizontal coordination with other policy subsystems and interdependence between parallel, dispersed, and unrelated policy domains in pursuit of a common policy outcome has been emphasized as a pervasive element of policy achievement. This is customarily defined as “policy integration” (Giessen, 2011a, b). The fundamental logic underlying policy integration is that cooperation among actors with different policies more effectively delivers the intended outcome than exclusively sectorial policymaking can. Unilateral policymaking, on the contrary, has the potential to undermine the objectives of parallel policy domains (Tosun & Lang, 2017). Hence, the context of the pandemic required intensely harmonized interventions between multiple sectors. For instance, the business sector was forced to adopt smart-working and flexible arrangements, even if such measures are normatively the exclusive domain of health care policy. The Ministry of Education in Italy, for example, urged the adoption of measures to suspend schooling and examinations. Furthermore, public transport authorities structurally revised their local delivery systems. By no means does policy integration necessarily facilitate the pursuit of joint policy objectives: the pursuit of policy integration requires coordinated institutional infrastructure to align the contributions of distinct domains. In a strongly decentralized system, however, the lack of overarching coordinating authorities has the potential to undermine cross-sector integration. Vertical coordination and policy integration are critical theoretical categories to analyze the COVID-19 early responses in Italy and are recognized as key variables for effective management (OECD, 2020).

The political outcomes of a crisis depend on the governance system’s capacity to meet public expectations. Furthermore, failure or success in crisis management has been interpreted as the gap between capacity and political legitimacy (Christensen et al., 2016). Therefore, governments must ensure output legitimacy by delivering measures perceived as appropriate, effective, and compliant with cultural norms. Primarily, sensemaking activities allow public authorities to understand the crisis, assess its magnitude, and deliver acceptable early-policy intervention (Boin et al., 2016). Sensemaking leads to decision-making strategies, based on the degree of threat, time urgency, and anticipation perceived by actors (Hermann & Dayton, 2009). The virus was new, and its devastating effects, unexpected. Policymakers faced an unprecedented challenge, and perceptions of the threat were based on fragmented information from China and the World Health Organization (WHO). Information from different epistemic communities of experts reached Italian policymakers in a confusing way. Each regional government acquired scientific policy advice from different virologists and epidemiologists; hence, the scientific community did not speak univocally.

Building a credible narrative for public opinion during transboundary crises is exceptionally problematic: “In fact, the rising number of actors increases the chances of contradicting messages, which may heighten fear and hamper cooperation” (Ansell et al., 2010, p. 200). Arguably, the relationship between coordination capacity and sensemaking is ambivalent: sense-making influenced coordination capacity by facilitating or harnessing early interventions and preparedness to manage the COVID-19 emergency. Therefore, the less adequate sensemaking is, the more likely the crisis will spiral out of control (Weick, 1988). Conversely, the degree of coordination within governance systems may enhance or deter a cohesive understanding of the crisis, depending on how multiple flows of information are channeled and filtered. The operational mechanisms that underpin the governance system during stable conditions, however, are completely undermined in the context of unprecedented crises (Ansell et al., 2010). The contingency plans created in the aftermath of the SARS virus in 2002, notably the regional pandemic plans that are analyzed in “Context: Italy” section, were never implemented in Italy. To gather political consensus, governments ought to create a compelling narrative and reassure citizens that public authorities are taking effective action to deal with the emergency. This typically requires a communication strategy to restore public trust and justify bureaucratic behavior. This point is emphasized in all regional pandemic plans. However, government detractors can undermine its political support, forcing governments to assume a defensive stance vis-à-vis aggressive criticism by media, political adversaries, and antagonist organizations. Failure to maintain legitimacy can result in political punishment, weakened reciprocity between actors or multiple levels of governance, hampered coordination, or any combination of these three outcomes.

The key outcome variable in this paper is the level of pre-crisis pandemic preparedness and anticipatory planning instruments put in place at national and regional scales to manage pandemics. How did regional governments implement their pandemic plans, as key policy instruments designed to enhance policy coordination and integration? The main hypothesis, in this paper, is that in a highly decentralized governance system, joint actions and collaboration between interdependent actors and levels of government is crucial for crisis management, and this emerged as the most significant governance criticality from early policy responses to COVID-19 in Italy.

The focus of “Context: Italy” section is the role of existing national and region-specific pandemic plans, as primary sources of evidence for crisis management policy capacity and coordination potential among interdependent actors. First, these operational tools were the earliest available instruments of response mechanisms to deal with the COVID-19 health care crisis, prescribing necessary assets, core strategies, and intervention competences. Second, the decentralized character of health care governance is embedded in these provisions, as the plans establish hierarchies, competences, and organizational arrangements between concurrent authorities. Literature suggests that since vertical and horizontal relations (institutional coordination and policy integration, respectively) among policy actors tend to favor formalized procedure rather than policymaking practice (Tosun & Lang, 2017), the level of operational and organizational compliance with the formulated pandemic plans is presumed to reflect intervention coherence, and the adequacy of pre-existing crisis coping mechanisms.

## Context: Italy

The Italian NHS was created by law in 1978. The reform was a response to the particularistic and clientelistic health care system of the 1960s and 1970s, based on one hundred health care funds with huge indebtedness. Law no. 833 of 1978 guarantees universal access and democratic participation to all Italian citizens. Moreover, the creation of the national health care system was concurrent with the definition of the new regional level of government (Mattei, 2006).

A landmark reform in 1992 ensured that regions had the authority to plan, organize, finance, and deliver health care services. Prior to 1992, these responsibilities were shared by the municipal administration and the central Ministry of Health. Although the active involvement of regions was not formally new, as it is embedded in the Constitution and in the 1978 Law, the ambition of the 1992 reform was to activate the formal powers of the regional government and tighten regional control on local health authorities and hospitals. Regional planning was the logical extension of national planning (Mattei, 2007).

The decree also established that regions had to fund everything that was not covered by their per capita national allowances. In the 1980s, the relationship between regions and the central state was conditioned by the lack of rational financial incentives (Ferrera, 1996). In addition to curbing spending and financing budget deficits, regions are expected to cover up to 80% of the costs of public hospitals with its share of national funding.

The most important stage in the development towards fiscal federalism is represented by Legislative Decree no. 56/00. It established the gradual shift of transfers from the center to the regions by abolishing the National Health Fund and the earmarked grant for the health care sector, based on a *quota capitaria* defined by the National Health Plan on a three-year basis.

The role of emergency powers was traditionally the remit of the central state until 1998, when the National Department of Civil Protection was reorganized to recognize a greater responsibility to regions, especially in the field of prevention and health care organization. During the COVID-19 crisis, the directly elected regional presidents frequently issued ordinances with emergency powers.

One of the most important instruments of government planning at central and local levels was the “pandemic plan.” The, Asian highly pathogenic avian influenza (HPAI) A(H5N1) virus and recommendations from the WHO prompted the design and adoption of the National Pandemic Plan (NPP) (*Piano nazionale di preparazione e risposta ad una pandemia influenzale*) in 2006. Its major objective was to identify, and issue early warnings of new cases of flu viruses, in order to take quick action at the start of the pandemic. The Ministry of Health takes responsibility for the policy coordination of all government actions to fight against pandemics. One of the key principles to improve policy capacity, according to the 2005 Plan, is to effectively coordinate all policy responses at national and regional levels. It provides, for instance, a comprehensive roadmap on what to do during Stage 1, identified as the “preparation and crisis prevention stage” before pandemics emerge. It calls for the improvement of preparation mechanisms, such as mapping hospital beds, ventilators, disinfection plans, stockpiling of personal protective equipment (PPE), analyzing triage arrangements and the possible movement of patients between health care organizations. It emphasizes the need to train a health care workforce, and personnel. The 2005 Plan provides clear and detailed guidelines on how to design and implement regional pandemic plans. Unfortunately, the 2005 Plan was never implemented. For instance, training exercises did not take place in any regions.

Three regional operational plans are examined in “Pandemic Plans” section.

## Pandemic Plans

The COVID-19 crisis had a strong territorial dimension in Italy, as presented in Table 1. Regions in the North were severely affected and suffered most infections and deaths registered in the country (ISTAT, 2020). Lombardy was overwhelmed by COVID-19 cases during the first wave, unlike other regions in the North (Veneto, for instance). The South was significantly less affected during the first wave. As regions in Italy share formal and legal responsibilities for pandemic prevention and control measures with the central government, coordination capacity is a criticality to jointly achieve the goals of protecting the population. Along the lines recommended by the NPP, first adopted in 2006, each regional government had to develop its own Regional Pandemic Plan (RPP) according to their demographic, socioeconomic conditions and health care organizational arrangements (Ministero della Salute, 2006). Regional governments acquired new responsibilities for crisis management and emergencies during the late 1990s. As the governance structure of the national Department for Civil protection changed from a centralized to a network and polycentric governance approach, regional governments, provinces, municipalities, and the voluntary sector became key stakeholders in crisis management. Regions were tasked with prevention and control functions during the pre-crisis contingency planning. In the field of crisis management, such as the COVID-19 pandemic, the central state held the main coordination responsibility. The Department of National Civil Protection supports regions with stockpiling of pharmaceuticals, and PPE, providing additional health care workforce and new hospitals, as necessary.

**Table 1:** Distribution of total infected cases and total deaths per macro area. *Source* ISTAT, Istituto Nazionale di Statistica (2020)

COVID-19 intensity	Geographic location	Cases (%tot)	Deaths (%tot)
High intensity	41 provinces (mainly Northern Regions)	75	82
Medium intensity	32 provinces (mainly Central Regions)	17	13
Low intensity	34 provinces (mainly Southern Regions, Isles)	8	5

When the COVID-19 pandemic struck Northern Italy in January 2020, the most updated NPP was the one adopted in 2006. The 2006 National Plan was a response to the WHO’s recommendation in 2005 to design pandemic plans in view of the SARS-2003 influenza. The Italian Ministry of Health designed comprehensive and detailed contingency planning instruments, indicating specific programs and actions of prevention and control that regional governments had to implement. An analysis of the NPP suggests that regional governments were given clear, specific guidelines on how to design local programs, which had to align with central national protocols. When the COVID-19 pandemic emerged in 2020, neither the National Plan nor the regional plans were used as governance tools. They were simply not activated nor used in the process of crisis management. The governance system was so overwhelmed by the pandemic that chaos and confusion reigned (Mattei & Del Pino, 2021). The 2006 National Plan (the only one available in 2020) recommended regional governments to implement clear communication strategies with the population, to establish clear protocols for the procurement and use of PPE, to create and implement training schemes for health care workers; and to monitor the available resources (hospital beds, diagnostic technologies). The key characteristics of selected regional plans for these dimensions of the national contingency planning follow. The purpose of this section of the paper is to present the regional articulations and different interpretations of nationally coordinated and mandated contingency planning.

Regional pandemic plans for Sardinia, Lazio, Veneto, and Lombardy, in place between 2006 and 2008 are analyzed. The analysis focuses on the three dimensions of comparison mentioned in “Context: Italy” section: communication strategies; PPE and resources; and training. Four regions that are most different in two respects were selected: first, they had vastly different epidemiological patterns during the first wave of the pandemic. Sardinia and Lazio were only minimally affected by the COVID-19 pandemic from January until June 2020, while Lombardy and Veneto were the most severe cases in Italy. Second, regional territories with extremely different socio-economic characteristics were chosen to avoid selection bias (Putnam, 1993). Regional governments vary in their administrative and governance capacities, ranging from the more developed Northern regions to Sardinia, with a lower organizational capacity. Each regional plan is now discussed and the key findings are presented.

### Veneto

In Veneto, the RPP prescribes step-by-step procedures aligned with the national guidelines, revealing a committed pursuit of coordination strategies with the central state (Giunta regionale del Veneto, 2007). Implementation responsibilities were expressly assigned to health care actors. Stockpiling and acquisition of PPE established that masks were to be supplied to health workers, and the general population during the initial stages of contagion. Effectively, most protocols and tasks were already in place upon adoption of the 2007 regional plan, as surmised in the implementation schedule annexed to the document. With the emergence of COVID-19, the standard of contingency planning was subject to a further consolidation, and management efforts were intensely focused on communication activities. The contribution of key scientists as strategic policy advisors to the regional government was paramount to decision-making processes, also due to the foresighted assumption that asymptomatic carriers were primary vehicles of infection. Considering this, the Regional Government extended testing to the entire population, far exceeding the groups indicated by the national protocol. While severe political controversies have arisen, the strategy was particularly successful at containing infections and fatalities compared to Lombardy; hence, fostering broad public support for the regional government.

### Lombardy

The situation in Lombardy was substantially worse in terms of numbers of cases and deaths. As previously suggested, the Lombardy provinces suffered almost as many infections and deaths as all other regions combined (ISTAT, 2020). Whilst the high population density and the areas intense economic activities facilitated the contagion, serious governance shortcomings hindered effective crisis management. The 2006 RPP appears extensive, broadly encompassing the areas indicated in the NPP. A clear mechanism of pre-crisis planning and monitoring is established through the designation of watchdog medics, and measures for the isolation of potentially infectious carriers are created (Consiglio regionale della Lombardia, 2006). However, the broader public health measures to manage high-intensity pandemic stages only consider the temporary closure of schools and the interdiction of mass gatherings. Compared to the national approach, Lombardy favored a hybrid strategy reliant on the deployment of adaptive, contextual and ad-hoc measures.

However, the premises of such design deteriorated as the spread of the virus exceeded the authorities’ capacity to introduce timely interventions. Similarly, with communication strategies, despite an elaborate formal procedure for their development in the plan, they are chiefly postponed at the onset of the crisis to deliver ad-hoc guidelines. In practice however, confusion about the virus hindered narrative-construction processes as late as the end of February 2020, when infections in the region were already rising exponentially. Furthermore, the pervasiveness of the contagion disrupted any development of a coherent governance system between different levels of government, causing vertical coordination underlaps between the regional government and the central state. Political conflict erupted between the President of the Lombardy Region, Mr. Attilio Fontana, and Prime Minister Giuseppe Conte, as both levels claimed ownership of the local emergency, thus delaying the curtailment in two high-priority towns in the Bergamo province, Nembro and Alzano Lombardo. Since institutional provisions allow intervention by the region or the central state, no action was taken to contain the virus in those cities which were not isolated (Comitato Tecnico Scientifico, 2020). As infections proliferated and casualties surged in these areas, political leadership diverted their efforts toward blame-games (ANSA, 2020a).

### Sardinia

The region of Sardinia’s experience with COVID-19 was fortunately limited during the first wave. It is crucial to comprehend that Sardinia enjoys two fundamental geostrategic advantages. As an island in the Mediterranean, it constitutes an isolated region where the economy hinges on summertime tourism. Therefore, the scarce circulation of people in winter may have inhibited the contagion. Although its urbanized areas host most inhabitants, cities and towns are not closely connected, further ameliorating curtailment. For pre-crisis pandemic arrangements established by the RPP, authorities opted for a close mirroring of national recommendations, featuring most elements prescribed in the NPP. The reference to PPE is worthy of attention: their employment is exclusively for hospitalized patients and does not extend to the entire population. A divergence from the NPP lies in the absence of specialized provisions to train the health workforce, although this shortcoming persists in most regions. In addition, communication strategies are emphasized; the purpose is to achieve high levels of internal coordination between policy response actors, provide information, and build trust with the citizenry. COVID-19’s impact in Sardinia was negligible, with a single spike of infections in late March at Sassari’s hospital, Santissima Annunziata, which constituted more than two thirds of total regional cases: 875 solely in Sassari of 1366 in Sardinia (Regione Autonoma della Sardigna, 2020). Interestingly, according to health care specialists, the cluster in Sassari emerged due to insufficient organizational capacity, with a lack of protective equipment, specialized training, and asymptomatic testing (ANSA, 2020b). Although outside the timeframe of this paper’s analysis, a further development of the Sardinian case ought to be advanced here, as the epidemiologic situation was completely reversed between July and August 2020. Since the relaxation of containment measures drew massive inflows of Italian and foreign tourists to the beaches, Sardinia became one of the main hubs for second-wave clusters in September.

### Lazio

In central Italy, the region of Lazio prioritized an operational approach to crisis management; its 2008 RPP sets an extensive framework of surveillance systems, prevention mechanisms and resource mobilization for the crises’ later stages. Coordination between actors emerges as a key element to ensure the continued provision of basic services. Furthermore, a strong early-warning mechanism to facilitate the identification of patients with infectious diseases in the absence of vaccines or established therapies, and mobilizing the Health Crisis Units in coordination with Hospital Lazzaro Spallanzani was considered important. This facility was the main center of containment for infected patients and hosted the two Chinese tourists identified as “Patient Ones” in late January (Carinci, 2020); this event alerted regional authorities timeously.

As of 2008, the plan envisioned the lockdown of socioeconomic activities, presenting briefings on quarantining and social distancing; the temporary closure of businesses and offices through lockdown measures was also contemplated. Guidelines on the use of PPE are limited in Lazio’s RPP, so detailed instructions were delivered later as ad-hoc Regional Recommendations adopted in early March 2020, to inform the health workforce and citizens about the correct use of masks and public hygiene measures (Regione Lazio, 2020b). The same rationale applied to train specialized health workers: while originally subordinated to the preparation of national-level instructors, the region employed distance-learning modules created ad-hoc with universities and the Veneto Region to supplement those provided by the National Institute of Health (“Istituto Superiore di Sanità,” the leading technical-scientific body of the Italian NHS) (Regione Lazio, 2020a) (Fig. 2).

## Concluding Discussion

Contingency plans are policy instruments that outline protocols, guidelines, and rules to follow in the event of a pandemic. Crisis planning is a significant policy making process that involves designing anticipatory measures and preparations to allow a governance system to respond swiftly in a crisis (Clarke, 1999; Hillyard, 2000). The central question of the article leading in to the hypothesis was: How did regional governments implement their pandemic plans, as key policy instruments designed to enhance policy coordination and integration? This article has offered an original analysis of the regional implementation of pandemic plans and investigated for the first time the approaches different regional governments in Italy adopted during the first wave of the COVID-19 pandemic in 2020. Thus far, only few studies have explored the role of pre-crisis contingency planning.

An observation of early responses to COVID-19 in Italian regions is useful to study the relationship between contingency pre-crisis planning instruments and crisis-management. The literature on transboundary crises has emphasized the complexity of interdependence between different levels of government and groups of specialized actors operating in overlapping policy spaces, encouraging a comparison of response systems to the same crisis as an insightful tool to elucidate these issues (Ansell et al., 2010). Similarly, the literature on crisis management stresses preparedness and preparation activities as mandatory functional tasks for public authorities during crises (Eriksson & McConnell, 2017; Hale et al., 2020; Weible et al., 2020).

The first wave of COVID-19 was an intense test of the Italian decentralized health care system, highlighting the non-linear relationship between formal pandemic plans and successful crisis management. The findings suggest that due to their fragmented institutional reach, highly decentralized systems, require additional effort to deliver an effective response strategy. Hence, ensuring policy alignment between different units of government and integrating actors from multiple policy sectors is essential to coordinate the collective crisis management effort. Preparedness planning aims to achieve that and prevent bad governance through the anticipation of administrative procedures, hierarchies, resources, and distribution of formal responsibilities. For Italy and its regions, such planning instruments were the NPP and the RPP’s; carefully studied and presented in great detail in the empirical section of the paper.

Our empirical analysis shows that some regional problems, during early crisis management, could have been minimized, had regional policy measures been better integrated with the anticipatory prescriptions of the NPP. The unavailability of protective masks, the delayed containment measures, and absence thereof, in Nembro and Alzano in Lombardy, and the infection clusters in Sassari’s hospital are all examples of inadequate and unsuccessful crisis management. Compliance with national directives and rigorous enactment of RPP’s provisions, however, would have been only marginally effective: pandemic plans at neither level received updates after the H1N1 influenza virus (swine flu) in 2009, whilst the scope of COVID-19 far exceeded the need for capacity envisioned therein. Despite these limitations, the implementation (albeit partial) of these policy instruments would have contributed to the development of purpose and learning during the management of pandemic crises (Hillyard, 2000). More research is necessary to understand the reasons for the lack of implementation of the regional pandemic plans in Italy, as some of them were very detailed, and rigorous.

Thus, one of the main findings is high levels of heterogeneity in implementing the NPP, as regional governments used discretion in setting out protocols, regulations, and crisis management guidelines. Given such marked differences, one could have predicted the further challenge to coordinated governance efforts in crisis management. Significantly, even where the RPP’s provisions were put in place, and pre crisis contingency planning was adequate and strong, that did not necessarily translate into effective crisis management. This finding demonstrates the validity of Erikson and McConnell’s argument on contingency planning (Eriksson & McConnell, 2017). In all four regional cases, pre-crisis successful planning did not lead to effective crisis management. Most organizations failed to follow the legal mandates and the plans. The Lombardy Region was overwhelmed by the COVID-19 pandemic and all the preparations and anticipation mechanisms were not implemented quickly enough to save lives.

Clarke has argued that pandemic plans can lay out good operational strategies, but then organizations fail to follow their mandates (1999). Successful pre-crisis contingency plans can make no difference. It seems, in Italy some regional governments fared better by improvising and adapting to uncertain conditions, and crafting innovative policy instruments of their own. Veneto was more effective than other regions by violating the national statutory protocols on track and trace. Lazio’s regional government too opted for this “learn as you go” approach, compensating for its poor pre-crisis planning mechanisms with ad-hoc legislation and intense horizontal policy integration with local health organizations. In this paper, we have not collected data with regards to sense-making and the perception of political and administrative elites, which possibly would have illuminated the process of strategic choices. Our analysis is not based on interviewing, due to the COVID-19 restrictions which impeded our direct on-site interviews and observations.

To conclude, Italy was the first European country to suffer from the impact of COVID-19 and may have been overconfident during the early stage of the crisis because its respective health systems ranked among the best worldwide (e.g., The Lancet Ranking), and Italy scored reasonably well in the Global Health Security Index (2019). The threat was also significantly downplayed in Italy until late February 2020. The empirical analysis conducted in this study indicates that some problems that arose during the crisis management of the pandemic could have been minimized if policy measures had been implemented rapidly and available policy instruments were used. Neither the general pandemic plans at the central government level, nor the existent preparedness plans for influenza and Ebola, anticipated the scope of an epidemic like COVID-19. However, they provided useful tools and protocols which were not implemented and completely ignored in many regions in Italy. Decentralized health care systems have advantages because they encourage innovation in regions that are trying to find solutions to the crisis and these answers can provide best practices, such as in Veneto in Italy. However, it is also likely that decentralized systems make coordination more complex. Central governments may be tempted to take on responsibility or avoid it. This happened in the case of Italy and made it difficult to be clear about who owns the crisis at the early stage, who should make the decisions and who should be punished or rewarded for it. The COVID-19 crisis has served as a learning experience about the limitations of pre-crisis planning and the role played by coordination capacity during crisis management. It has, nevertheless, helped to identify some aspects of pandemic management that require improvement. Future research is needed on the consequent pandemic waves, occurring in Italy from December 2020 and April 2021, in order to analyze the extent of policy learning from earlier experiences, and assess any changes to the coordination mechanisms here discussed in the paper.
